# PP80 Experience Of The Health Technology Assessment Committee At Hospital Italiano De Buenos Aires

**DOI:** 10.1017/S0266462325102985

**Published:** 2025-12-29

**Authors:** Ana Paula Gomez, Maria Florencia Filloy, Mariana Andrea Burgos, Karina Elena Alvarez, Gabriela Buela, Luis Angel Di Giuseppe

## Abstract

**Introduction:**

Innovation in medications creates challenges for access to health care, making the rational use of drugs essential. The Health Technology Assessment Committee (HTAC) help to adapt technologies to hospital settings and align recommendations with institutional needs. We aimed to describe the role of the HTAC in a high-complexity hospital by quantifying the requests received and approved.

**Methods:**

The study was conducted at the Hospital Italiano de Buenos Aires. The primary objective was to provide a descriptive analysis of the functioning of the HTAC. The secondary objective was a retrospective observational study of the quick reports produced by the HTAC between January 2017 and December 2023. Variables analyzed included the total number of drug evaluation requests received and the proportion of requests incorporated or not incorporated annually during the study period.

**Results:**

The HTAC at our institution began in 2011, initially consisting of one physician, one pharmacist, and one administrative staff member. It now includes two coordinators and 11 evaluators.

The drug incorporation process starts with a request from the prescribing service, followed by a literature review and specialist meetings. A technical report is produced that covers efficacy, safety, costs, and final recommendations. During the study period, 111 drug incorporation requests were received, with 90 (81.1%) being included in the formulary. In the 2022 to 2023 biennium, 50 requests were received, representing 45 percent of the total requests during the study period.

**Conclusions:**

Standardizing the HTAC’s structure was essential for managing the increasing number of new drug approvals. Adapting evidence on new molecules to the hospital setting was crucial to support recommendations for inclusion in the therapeutic formulary and to optimize the use of technologies, enabling more efficient and structured decision-making.

